# Multi-Layer Application of Self-Etch and Universal Adhesives and the Effect on Dentin Bond Strength

**DOI:** 10.3390/molecules24020345

**Published:** 2019-01-18

**Authors:** Anna Zecin-Deren, Jerzy Sokolowski, Agata Szczesio-Wlodarczyk, Ireneusz Piwonski, Monika Lukomska-Szymanska, Barbara Lapinska

**Affiliations:** 1 Department of General Dentistry, Medical University of Lodz, 251 Pomorska St., 92-213 Lodz, Poland; anna.zecin@stud.umed.lodz.pl (A.Z.-D.); jerzy.sokolowski@umed.lodz.pl (J.S.); barbara.lapinska@umed.lodz.pl (B.L.); 2 University Laboratory of Materials Research, Medical University of Lodz, 251 Pomorska St., 92-213 Lodz, Poland; agata.szczesio@umed.lodz.pl; 3 Department of Materials Technology and Chemistry, Faculty of Chemistry, University of Lodz,163 Pomorska St., 90-236 Lodz, Poland; ireneusz.piwonski@chemia.uni.lodz.pl

**Keywords:** dental bonding system, adhesion, self-etch adhesive, universal adhesive, bond strength, dentin, scanning electron microscopy, adhesive layer

## Abstract

Contemporary self-etch and multi-mode adhesives were introduced to ensure a fast and reliable bonding procedure. Yet, in terms of bond strength and stability they failed to perform as well as two-bottle, etch-and-rinse adhesives, which remain the gold standard in terms of durability. The purpose of this study was to assess the shear bond strength (SBS) of dental adhesives to dentin with different application protocols. Two self-etch (Adper Easy One and Xeno V) and two multi-mode adhesives (Single Bond Universal and Prime&Bond One Select) were used in the study. The highest SBS was obtained for Single Bond Universal applied in three layers, while the lowest, for Xeno V applied in one layer. Other tested adhesives obtained the highest SBS when applied in three layers. For all tested adhesives, multi-layer application resulted in an increase in adhesive layer thickness, as observed in SEM. The increased thickness of the adhesive layer produced by triple application of unfilled adhesives corresponded with higher SBS values. The present study showed that using triple adhesive layers with simplified adhesive systems can be recommended to improve their performance. Due to differences in the composition of self-etch and universal adhesives, the exact application protocol is product dependent.

## 1. Introduction

There are many dental adhesives available on the market. They can be classified in three categories regarding bonding techniques to the dental substrates: the etch-and-rinse (ER, formerly known as total-etch), self-etch (SE) or universal (multi-mode, MM) systems [1,2]. The ER strategy involves orthophosphoric acid etching of both enamel and dentin, which results in micromechanical retention of resin to the tooth structure. Enamel is a tissue consisting mainly (in 96 wt%) of a hard, solid crystalline structure—hydroxyapatite (HAp). When phosphoric acid is applied, HAp becomes selectively dissolved, creating micro- and macro-porosities that enable the infiltration of resin monomers. After polymerization, the resin becomes interlocked within the porosities [3]. It was proven possible to achieve an efficient, reliable bond to enamel with the ER technique [4] and it is considered the gold standard in terms of durability in adhesive dentistry [5]. Bonding to dentin however, remains a challenge, mainly due to its more organic composition. Dentin is a biological composite of HAp (50% vol.) that envelops collagen (30% vol.), and water (20% vol.). While etching dentin, the acid demineralizes intertubular dentin, which results in exposing superficial collagen network [6]. The network is infiltrated with the adhesive resin, which leads to formation of a hybrid layer, responsible for the bond between the resin and dental tissues [7,8,9]. To ensure optimal conditions, the demineralized dentin must be kept moist in order to prevent collagen fibrils from collapsing. Simultaneously, dentin must not be too wet as excessive moisture will prevent full impregnation of collagen fibrils with resin monomers [3,10].

To resolve the problem of optimal wetness of dental substrate as well as to reduce the technique sensitivity associated with ER adhesives and shorten the manipulation time, adhesives using self-etch strategy (SE) were introduced. They contain acidic hydrophilic monomers that demineralize dentin and simultaneously infiltrate it. Due to the fact that the pH of these monomers is higher than that of orthophosphoric acid, self-etch adhesives (SEA) demineralize dentin more superficially than etch-and-rinse adhesives (ERA) do [11]. They do not remove the smear layer, but use it as a bonding substrate and leave residual smear plugs in the dentin tubules. This seems to be responsible for the lack of postoperative sensitivity associated with ERA. Contrary to expectations, the first SEAs did not perform well in the clinical setting, presenting a number of drawbacks [4,11]. These included decreased immediate and long-term bond strength [12,13], increased interfacial nano-leakage after aging [14], enhanced water sorption (2-hydroxylethyl methacrylate or HEMA containing systems) [15,16], the monomer from the solvent phase separation (HEMA free systems) [17], difficulty in the optimal evaporation of the solvent and shelf-life problems [18]. Moreover, due to their higher pH, SEAs were unable to etch enamel to the same depth as phosphoric acid did, resulting in lower enamel bond strength than that achieved with phosphoric acid etching [19,20].

Further advances in adhesive dentistry involved introducing new molecules into the composition of adhesives: 10-MDP (methacryloyloxi-decyl-dihydrogen-phosphate), 4-MET (4-methacryloxyethyl trimellitic acid) and phenyl-P (N-Phenyl-p-phenylenediamine). They were named functional monomers and were speculated to bond chemically to calcium in HAp [21]. Among these molecules, MDP was reported to possess the strongest chemical adhesion potential to HAp, forming stable calcium salts due to the process of nano-layering [22]. It was also proved that pre-etching enamel with phosphoric acid prior to the application of SE adhesives improved enamel–resin bond strength [23,24]. A new family of adhesive systems was launched on the basis of these outcomes. They are called multi-mode or universal adhesives due to their versatile indications for use. They can be used as self-etch (SE) adhesives, etch-and-rinse (ER) adhesives, or as SE adhesives on dentin and ER adhesives on enamel (a technique commonly referred to as “selective enamel etching”, SEE) [25]. Some universal adhesives contain silane in their formulation, potentially eliminating the silanization step when bonding to e.g., glass ceramics or resin composites. Nonetheless, it is known that adhesives with a simplified application procedure are associated with lack of proper marginal integrity, marginal discoloration and the loss of retention of fillings adhesively bonded to dentin [4].

Many authors have suggested modifying the application method of simplified, one-bottle SE adhesives in order to improve their properties [26,27,28,29,30]. The alterations included creating a thicker adhesive layer, which was expected to effectively balance the stress at the composite material-tooth interface. The stress was generated by polymerization shrinkage, mechanical load or temperature changes [28,31]. It was found that the greater the thickness, the more elastic the adhesive layer, hence the deformation of resin suppressed the stress [32]. Moreover, Pashley et al. [33] observed that the additional application of resin could seal the non-polymerized oxygen inhibition layer, increasing its conversion, enabling more complete polymerization, and therefore, leading to stronger adhesion to dentin.

The purpose of the study was to determine the effect of multiple coatings of all-in-one self-etch and multi-mode adhesives on dentin bond strength.

## 2. Results

### 2.1. SEM and EDS Analysis

**Figure 1:**
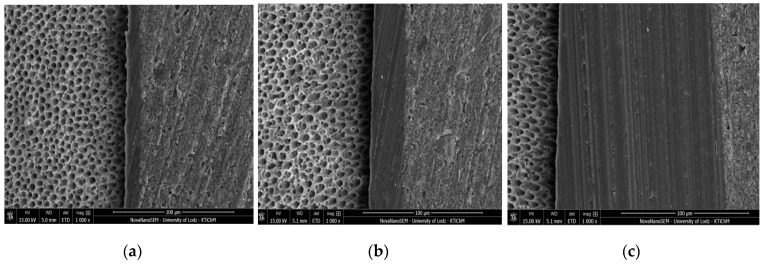
SEM images of adhesive layers obtained by application of Adper Easy One: (**a**) one layer; (**b**) two layers; (**c**) three layers; mag. 1000x.

**Figure 2:**
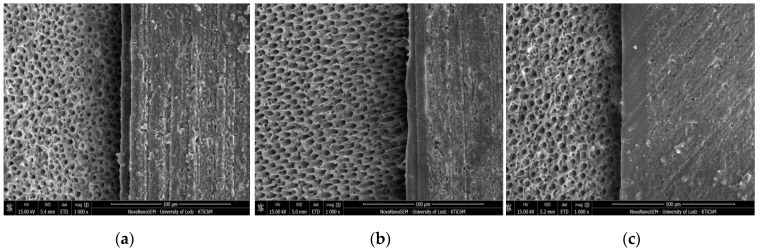
SEM images of adhesive layers obtained by application of Xeno V: (**a**) one layer; (**b**) two layers; (**c**) three layers; mag. 1000x.

**Figure 3:**
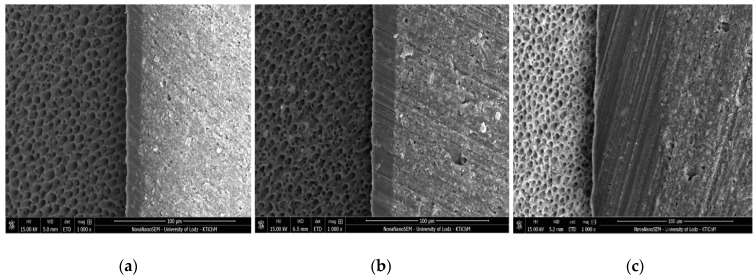
SEM images of adhesive layers obtained by application of Single Bond Universal: (**a**) one layer; (**b**) two layers; (**c**) three layers; mag. 1000x.

**Figure 4:**
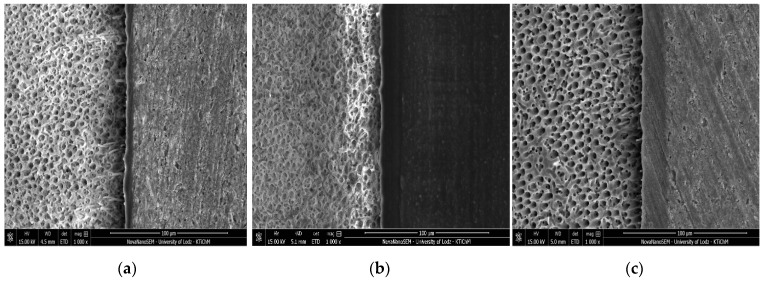
SEM images of adhesive layers obtained by application of Prime&Bond One Select: (**a**) one layer; (**b**) two layers; (**c**) three layers; mag. 1000x.

Figure 1, Figure 2, Figure 3 and Figure 4 present SEM images of dentin-resin interface obtained after application of the tested adhesives in one, two or three layers. SEM analysis showed that multiple adhesive coatings produced an increase in adhesive layer thickness for all the adhesive systems tested.

The mean values of adhesive layer thickness measured for the tested adhesives are presented in Table 1. The thinnest adhesive layer was obtained for Xeno V applied in 1 layer (control) and the thickest was for Single Bond Universal applied in 3 layers.

**Figure 5:**
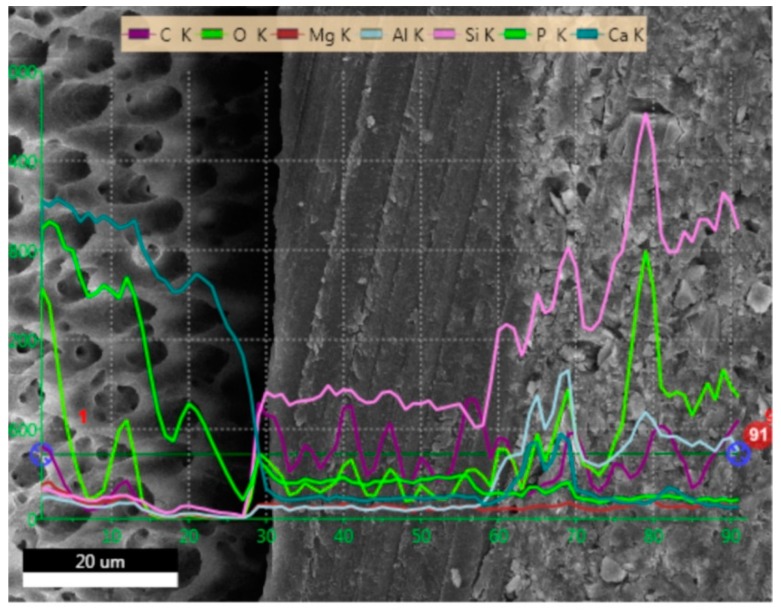
EDS line scan signals of individual elements present at dentin-resin interface obtained for Adper Easy One applied in two layers.

**Figure 6:**
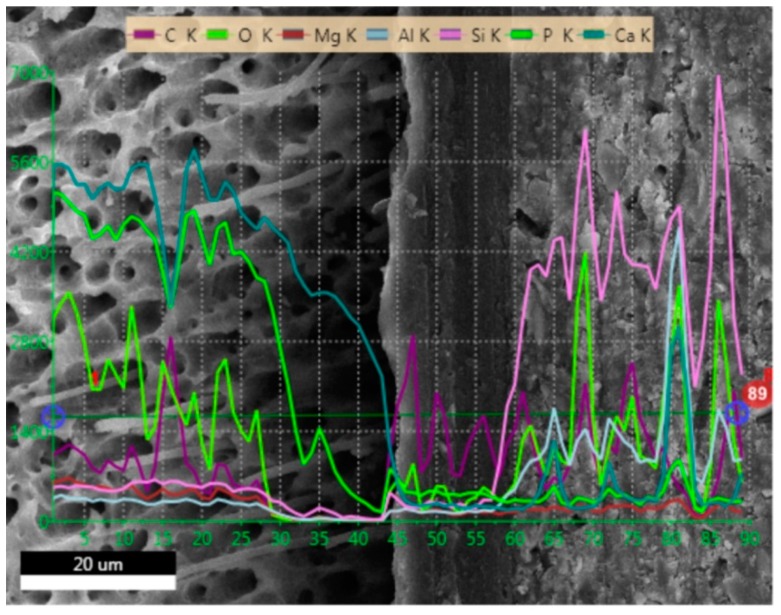
EDS line scan signals of individual elements present at dentin-resin interface obtained for Xeno V applied in two layers.

**Figure 7:**
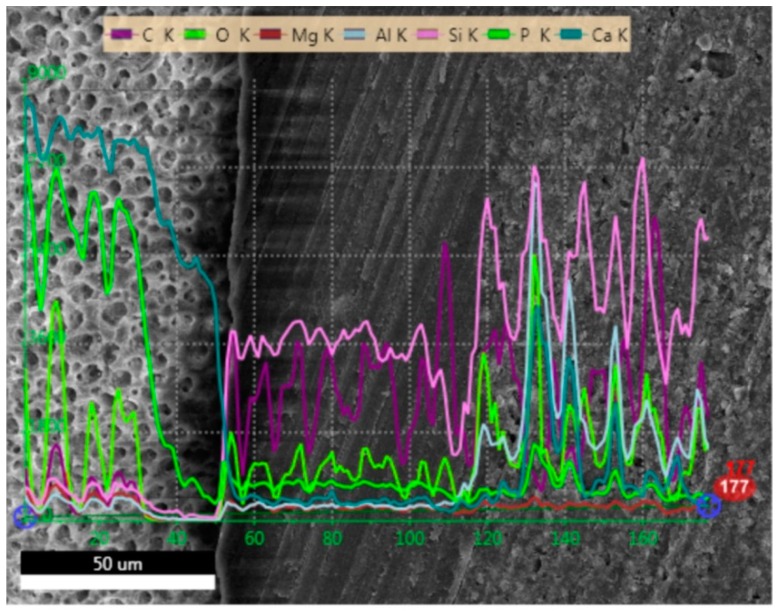
EDS line scan signals of individual elements present at dentin-resin interface obtained for Single Bond Universal applied in two layers.

**Figure 8:**
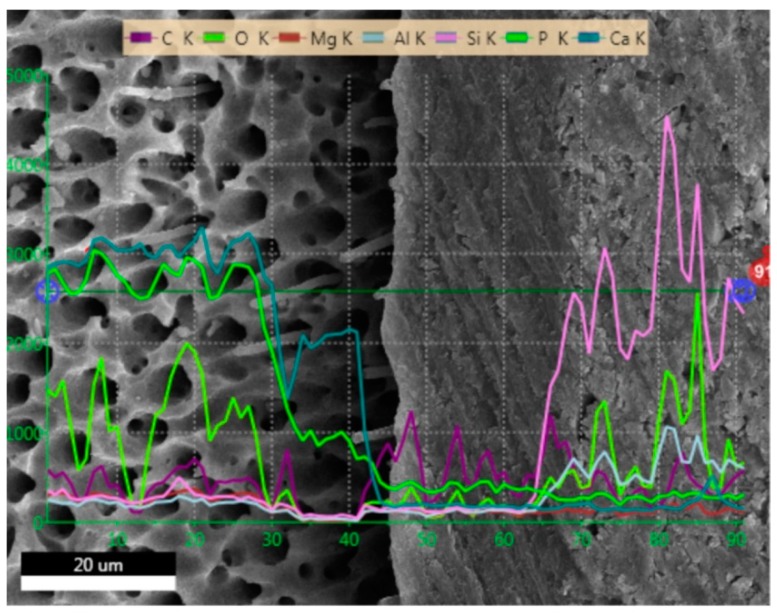
EDS line scan signals of individual elements present at dentin-resin interface obtained for Prime&Bond One Select applied in two layers.

The EDS (Energy Dispersive Spectroscopy) analyses of the dentin-resin interface are presented in Figure 5, Figure 6, Figure 7 and Figure 8. SEM/EDS analysis proved that the concentration of calcium and phosphorus, high in dentin, immediately dropped at the dentin-resin interface. Within the adhesive layer an increase in carbon was observed for all of the tested adhesives, and could be attributed to the fact that resin monomers contained that element. Adper Easy One and Single Bond Universal samples (Figure 5 and Figure 7) showed the presence of silicon and aluminum in the adhesive layer, as well as in dentin, however in smaller concentration. This is due to the addition of nanofiller in their composition, and further confirms that resin tags were produced by these adhesive systems. EDS analysis showed an increase of silicon and aluminum in the composite material in all specimens tested, indicating the presence of an inorganic filler in the material.

### 2.2. Shear Bond Strength

The results of the shear bond strength (SBS) test obtained for each bonding system are presented in Table 2.

**Figure 9:**
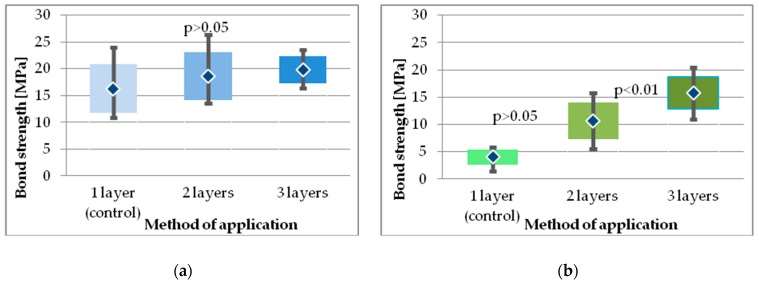
SBS results for multi-mode adhesives: (**a**) Single Bond Universal; (**b**) Prime&Bond One Select.

Among all tested adhesives, the highest SBS in all study groups was obtained for Single Bond Universal, a multi-mode adhesive (Table 2). The increase in number of layers up to three resulted in higher SBS for Single Bond Universal adhesive (from 16.30 ± 4.59 up to 19.8 ± 2.59 MPa), however, there was no statistical difference between the study groups (Figure 9a).

Other tested adhesives obtained the highest SBS when applied in three layers, but all these values were still lower than those obtained for Single Bond Universal applied in one layer (Table 2).

**Figure 10:**
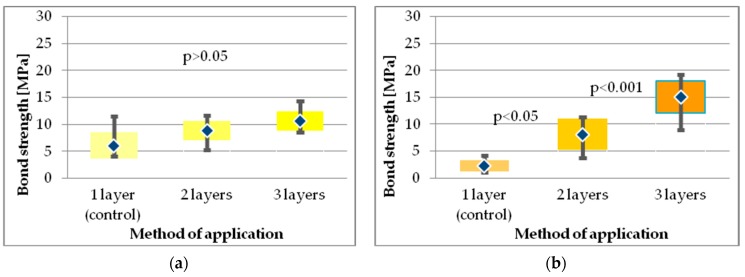
SBS results of self-etch adhesives: (**a**) Adper Easy One; (**b**) Xeno V.

The lowest values of SBS were observed for SE adhesive, Xeno V applied in one layer (2.21 ± 1.05), however, the bond strength significantly increased when the adhesive was applied in two or three layers (Table 2, Figure 10b).

The SBS of Adper Easy One (SEA) applied in one layer was higher than for Xeno V adhesive in the respective study group, although increasing the number of coats did not significantly affect the bond strength value of the Adper Easy One (Figure 10a). Both tested SE adhesives applied in two layers achieved comparable SBS results, while application of three layers resulted in a significantly higher SBS for Xeno V adhesive than for Adper Easy One.

Also, when compared with Single Bond Universal (MMA), Prime&Bond One Select showed lower bond strength in all respective groups.

Kruskal-Wallis ANOVA revealed a significant interaction between factors (type of adhesive, number of layers). For all of the adhesives, each additional layer resulted in an increase in bond strength. However, Dunnett’s post hoc test revealed that the difference in bond strength obtained after application of two or three layers was statistically significantly higher than for the control group only for Xeno V and Prime&Bond One Select (Figure 9b and Figure 10b).

## 3. Discussion

A reliable and durable bond to both enamel and dentin is the key to the clinical success of adhesive (restorative) dentistry. This prevents complications such as marginal degradation of the adhesive interface or secondary caries, which are the main reasons for the replacement of composite fillings [34]. Moreover, it enables minimally invasive intervention during cavity preparation. However, contemporary SEAs and MMAs fail to provide equally effective bonding performance to dentin as ERA.

It has been proven that the hybrid layer is responsible for adhesive-dentin bond strength [7,35]. It consists of collagen fibrils enveloped by resin. In order to generate high bond strength, the bonding agent must penetrate uniformly through the collagen system and be effectively polymerized. In the present study, several coats of adhesive were applied in order to determine, whether increased thickness of the adhesive layer may contribute to more reliable bonding to dentin. If that layer is too thin, the polymerization process may not fully proceed due to the oxygen inhibition phenomenon [36]. Also, due to the osmotic properties of hydrophilic monomers, water penetration through the insufficiently polymerized resin may be stimulated. This gives rise to so-called “water droplets” and “water trees”, and the resulting nano-leakage can weaken the resin bond to dentin [10].

In the present study, for Xeno V and Prime&Bond One Select a noticeable increase in adhesive layer thickness, as detected between the control and 3 layers group, was observed along with significantly higher SBS. Such correspondence was not found for Single Bond Universal and Adper Easy One. In case of these adhesives, the increase in bond strength between the control and the 3 layers group was not statistically relevant, even though the thickness of the adhesive layer was significantly higher.

The application of one layer of Adper Easy One resulted in higher SBS compared to Xeno V. Adper Easy One contains MHP (methacrylohexyl phosphate) and Vitrebond™ Copolymer, both of which could potentially create a chemical bond with hydroxyapatite [37] and seem to be responsible for improved bond strength. The presence of nanofiller in Adper Easy One may also have enhanced SBS when compared to Xeno V (containing no filler). Many authors suggested that including micro- or nanofiller in the composition of adhesives increases their viscosity, which contributes to creating a thick, reinforced hybrid layer [38,39]. In the present study, the adhesives containing filler in their composition (Single Bond Universal and Adper Easy One) generally produced a thicker adhesive layer than the non-filled ones (Prime&Bond One Select and Xeno V).

The comparison of SBS results of the tested SEAs showed that the bond strength of Adper Easy One to dentin was less affected by the number of layers than Xeno V. Each additional layer improved the SBS of Adper Easy One to dentin, but the differences were not statistically relevant. This was probably due to the fact that one layer of Adper Easy One produced sufficient adhesive layer thickness thanks to the presence of nanofiller in its composition. The adhesive layer thickness obtained by one layer of Adper Easy One was almost twice as high as for Xeno V in the respective study group (13.31 ± 0.43 µm vs. 7.40 ± 0.82 µm). It was proved in previous studies that an adhesive layer of an adequate thickness can provide more efficient stress resistance of adhesive systems [40]. Additionally, the components of Adper Easy One adhesive, such as MHP and Vitrebond™ Copolymer form a complex with calcium ions, resulting in a chemical bond with hydroxyapatite [41]. Therefore, each additional layer of Adper Easy One enhanced the bond strength, but did not contribute to a statistically significant increase in SBS to dentin. The application of several layers of Xeno V significantly improved its bonding performance to dentin. Each additional layer contributed to an increase in the adhesive layer thickness, confirmed in SEM observations, and supposedly to more complete polymerization and therefore higher SBS. These results corresponded with previous literature findings [42], which reported that the application of only one coat of Xeno V was not enough to create a sufficiently thick hybrid layer.

When Adper Easy One and Xeno V were applied in three layers, Xeno V obtained higher SBS than Adper Easy One. Therefore, it may be concluded that it is not the presence of nanofiller itself that leads to higher SBS results, but more the quality of the adhesive layer and its optimal thickness. Also, the presence of MHP in the composition of Adper Easy One did not seem to contribute to a substantial improvement in bond strength to dentin when compared to Xeno V applied in three layers. The resulting adhesive layer thickness probably achieved its optimal level for Xeno V and was less favorable for Adper Easy One performance.

Prime&Bond One Select was another unfilled adhesive included in this study. Mean SBS values increased with each additional layer of the adhesive, although the difference was statistically significant only between application of one and three layers. The results in the one-layer group for Prime&Bond One Select (4.02 MPa) were higher than for Xeno V (2.21 MPa), which may be attributed to the presence of acrylic acid in the composition of Xeno V. Both adhesives contain “inverse” methacrylic ester functions, which could possibly produce chemical interaction between monomers and dentin [43]. However, further research is necessary to confirm whether this phenomenon occurs at all. Both Adper Easy One and Single Bond Universal showed higher SBS in control groups compared to Prime&Bond One Select, which can probably be attributed to the presence of nanofiller and functional monomers in their composition.

When Prime&Bond One Select was applied in two and three layers, the SBS improved, probably due to an increase in the adhesive layer thickness, confirmed by SEM observations. However, when compared to Single Bond Universal, another multi-mode adhesive used in this study, Prime&Bond One Select obtained lower SBS to dentin in each respective group. According to the manufacturer’s instructions, Prime&Bond One Select is suitable for all etching techniques: ER, SE and SEE. However, further analysis of its composition led to the conclusion that it was comparable to Xeno V (Table 3), making it an all-in-one product with widened indications for use. The adhesive layer thickness of Prime&Bond One Select observed in SEM was also comparable to that of Xeno V in all respective groups.

Single Bond Universal, on the other hand, contains nanofiller in its composition. This might contribute to the production of a thicker adhesive layer than that of Prime&Bond One Select, which was confirmed by SEM observations in the present study. Also, Single Bond Universal includes MDP monomer, which shows higher intensity of nano-layering than the MHP monomer [37] present in Adper Easy One, another adhesive containing nanofiller tested in the study. MDP consists of a phosphoric-acid functional group, which is speculated to interact chemically with hydroxyapatite crystals, forming stable calcium-phosphate and calcium-carboxylate salts, along with only a limited surface-decalcification effect. According to the adhesion–decalcification (AD) concept, the less soluble the calcium salt of the acidic monomer, the more intense and stable is the molecular adhesion to the HAp-based substrate [41,44]. MDP also contains a methacrylate polymerizable group responsible for curing potential and a 10-carbon chain group to separate both other active groups [45]. The carbon spacer influences monomer flexibility, solubility, wetting, and the hydrophobicity-hydrophilicity balance [46]. This additional chemical interaction between MDP and dentin is thought to be responsible for the substantial improvement in bond durability [24,47,48]. This may also be the reason that Single Bond Universal had the highest mean SBS compared to all other adhesives used in this study.

The application of one and two additional layers of Single Bond Universal improved its SBS to dentin, but the differences were not statistically relevant. At the same time, a significant increase of adhesive layer thickness was observed. This may indicate that the adhesive layer thickness itself is not responsible for the SBS of Single Bond Universal to dentin as much as the MDP content in the adhesive’s composition. During the application of the second and the third layer, the concentration of MDP in the first layer did not increase, because the adhesive has already been polymerized. In order to further enhance the bond strength of Single Bond Universal to dentin it may be advisable to apply it two or three times and polymerize it after the application. Such protocol could contribute to enhancing the concentration of MDP.

## 4. Materials and Methods

### 4.1. Sample Preparation

Human molars that were intact, non-carious, non-restored, and extracted due to periodontal or orthodontic reasons were collected. The teeth were stored in 0.5% chloramine solution and used within 3 months of extraction. Using a low-speed diamond saw (precision micro-cutting machine, Mecatome T210 Prezi, France) under water lubrication, the crowns were separated from the roots and then the crowns were sectioned mesio-distally into two parts: buccal and lingual. Next, the dentin surface was ground under water coolant with 180-grit SiC followed by 600-grit silicon carbide paper to create a smear layer of clinically relevant thickness. The samples’ surfaces were examined under 10x magnification (optical microscope, Optilion PICO LED, Seliga Microscopes, Lodz, Poland) to ensure that they were free of retained enamel. The samples (*n* = 168) were randomly divided into four groups (*n* = 42), depending on the adhesive used.

### 4.2. Research Model

The tested adhesives (Table 3) were applied on the dentin surface in one, two or three layers.

In study group 1 (control group), the application of a single layer was performed following the manufacturers’ instructions. Using a disposable microbrush, the adhesive was rubbed into the dentin surface for 20 s. Next, a gentle stream of air was directed over the adhesive for 5 s, until it no longer moved and the solvent evaporated, creating a uniform, slightly shiny film. The adhesive was light-cured for 20 s.

In study group 2, each layer of the adhesive was applied in the abovementioned manner and subsequently polymerized. The procedure was repeated. Two separately polymerized layers of adhesive were obtained.

In study group 3, each layer of the adhesive was applied in the abovementioned manner and subsequently polymerized. The procedure was then repeated two times. Three separately polymerized layers of adhesive were obtained.

### 4.3. SEM and EDS Analysis

For each study group, 3 dentin samples with adhesives applied according the research model were prepared. The resin-dentin interface was exposed and polished using 180, 600 and 1000-grit silicon carbide paper under water coolant. The specimens were demineralized with a 37% orthophosphoric acid solution for 1 min and rinsed with distilled water for another minute. They were immersed in 5% NaOCl solution for 5 min to remove the organic debris. The solution was replaced 4 times after 1 min in order to prevent deactivation of NaOCl. After being rinsed with distilled water, the specimens were dehydrated in ascending ethanol concentrations (50, 70, 90 and 95% for 20 min each and 100% for 1 h), and then transferred to a critical point dryer for 30 min. All specimens were then gold sputter coated and the surfaces were examined in a scanning electron microscope (SEM, FEI Nova NanoSEM 450, FEI, Hillsboro, OR, USA) under 1000x and 2000x magnification. The adhesive layer thickness was measured based on the SEM images. Also, the chemical analysis of the dentin-resin interface was performed using energy dispersive spectrometry (EDS, EDAX/AMETEK, Materials Analysis Division, Model Octane Super, Mahwah, NJ, USA).

### 4.4. Shear Bond Strength

For the shear bond strength test, the dentin samples (*n* = 132) were embedded in PMMA in PCV tubes. In order to prevent temperature rise during polymerization of PMMA, which may have a negative effect on dental tissues, the samples were placed in cold water as soon as the chemical reaction began. After PMMA was set, the samples’ surfaces were ground under water coolant with 180-grit followed by 600-grit SiC grinding papers to create a smear layer of clinically relevant thickness. The samples’ surfaces were examined under 10x magnification (optical microscope) to ensure that they were free of retained enamel. The samples were randomly divided into four groups (*n* = 33) depending on the adhesive used. All adhesives used in the study are presented in Table 3.

The adhesives were applied on the dentin samples according to the research model presented above, and polymerized with an LED curing lamp (Elipar™ S10 LED Curing Light, 3M ESPE, Germany). Flowable composite (Flow-Art, Arkona, Poland) was then applied with the use of a silicone ring with the inner diameter of 3 mm and height of 4 mm. It was applied and light-cured in 2 increments. After 24 h storage in saline, the samples were shear loaded to fracture at 2 mm/min crosshead speed using a universal testing machine of 20 kN maximum load cell capacity (Zwick-Roell Z005, Zwick-Roell, Germany), with a gap distance between the crosshead and substrate less than 1 mm.

### 4.5. Statistical Analysis

Statistical analysis of shear bond strength (SBS) and adhesive layer thickness results was performed. To detect differences in bond strength and the thickness of adhesive layer between study groups regarding number of coats (1, 2, and 3) a Kruskal-Wallis ANOVA test was used. Dunnett’s post-hoc test was applied at *p* < 0.05 to detect which means were statistically different from each other. To compare each group with control, Mann-Whitney’s and Dunnett’s post-hoc test were used at *p* < 0.05.

Pairwise comparison of the adhesives (SE, MM) within the same group (1, 2 or 3 coats) was performed using Mann-Whitney’s test.

A level of *p* < 0.05 was considered statistically significant. All the statistical procedures were carried out using STATISTICA 10 (version 10, Publisher: StatSoft, Poland,).

## 5. Conclusions

Considering the limitations of the present study, producing double or triple adhesive layer with simplified adhesive systems can be recommended to improve their performance. However, one should bear in mind that due to differences in the composition of self-etch and so-called universal adhesives, the exact application protocol is product dependent. In order to achieve maximum efficiency, a specific protocol for each adhesive should be determined.

## Figures and Tables

**Table 1:** Adhesive layer thickness [µm] (mean values ± standard deviation) of the tested adhesives.

Adhesive	Method of Application		
	One Layer (Control Group)	Two Layers	Three Layers
Adper Easy One	13.31 ± 0.43 ^a^	26.15 ± 2.99	57.02 ± 48.16 ^a^
Xeno V	7.40 ± 0.82 ^b^	16.32 ± 1.05	18.62 ± 2.17 ^b^
Single Bond Universal	11.86 ± 1.68 ^c^	17.73 ± 1.40	72.33 ± 9.85 ^c^
Prime&Bond One Select	7.89 ± 1.09 ^d^	16.47 ± 0.70	18.79 ± 1.36 ^d^

Within tested adhesives, means followed by the same superscript letters in row indicate statistically significant differences.

**Table 2:** Shear bond strength [MPa] (mean values±standard deviation) of the tested adhesives.

Adhesive	Method of Application		
	One Layer (Control Group)	Two Layers	Three Layers
Adper Easy One	6.06±2.43 ^ab^	8.82±1.73 ^e^	10.58±1.79 ^h^
Xeno V	2.21±1.05 ^ac^	8.11±2.93 ^f^	15.00±2.99
Single Bond Universal	16.30±4.59 ^bcd^	18.60±4.42 ^efg^	19.80±2.59 ^h^
Prime&Bond One Select	4.02±1.42 ^d^	10.64±3.41 ^g^	15.80±2.91

For the tested adhesives, the means followed by the same superscript letters in each column indicate statistically significant differences.

**Table 3:** Dental adhesives used in the study.

Adhesive	Manufacturer	Composition	Mode of Etching
Adper™ Easy One	3M ESPE, Germany	MHP Phosphate Monomer, Dimethacrylate resins, HEMA, Vitrebond ™ Copolymer, Nanofiller, Ethanol, Water, Initiators	SE
Xeno V	Dentsply DeTrey GmbH, Germany	Bifunctional acrylic amides, Acrylamido alkylsulfonic acid, “inverse” functionalized phosphoric acid ester, Acrylic acid, Camphorquinone, Coinitiator Butylated benzenediol, Water, tert-Butanol	SE
Single Bond ™ Universal	3M ESPE, Germany	MDP Phosphate Monomer, Dimethacrylate resins, HEMA, Vitrebond ™ Copolymer, Nanofiller, Ethanol, Water, Initiators, Silane	MM ^1^ (universal)
Prime&Bond One Select	Dentsply DeTrey GmbH, Germany	Bifunctional acryl resin with amide functions, Acryloylamino alkylsulfonic acid, “inverse” functionalized phosphoric acid ester, Camphorquinone, Coinitiator Butylated benzenediol, Water, tert-Butanol	MM ^1^ (universal)

^1^ MM = ER&SE&SEE.
